# Modern Medico-Psychology and Psychiatry

**Published:** 1894-05-12

**Authors:** T. S. Clouston

**Affiliations:** Lecturer on Mental Diseases, Edinburgh University, and Physician Superintendent Royal Asylum, Morningside


### Modern Medico-Psychology and Psychiatry.

By T. S. Clouston, M.D., Lecturer on Mental Diseases, Edinburgh University, and Physician Superintendent Royal Asylum, Morningside.

### V.—The Men Who Have Made Modern Psychiatry.

No legislative measures and no mere institutions, however perfect, would have built up modern psychiatry, or could have revolutionised the treatment of the insane in fifty years. This great work needed men even more than measures. It is quite impossible to sketch in such a series of articles as this, even in faint outline, the careers and the characteristics of the men who have accomplished this. They may be divided into four groups. The first group consisted of the pure medico-psychologists. They collated and co-related mental and physiological facts, normal and pathological, endeavouring to bring both under laws that would apply to, and so far explain their association. They had the philosophic spirit and their writings had a large and somewhat theoretical tone. Feuchtersleben, of Vienna, in his "Medical Psychology," fairly broke ground in this department, and, with a truly German insight, showed the capabilities of this new branch of science. The next great medico-psychologist was Laycock, of Edinburgh, who, beginning by studying the "Nervous Diseases of Women," ended by his monumental work on "Mind and Brain." As my predecessor in the lectureship in the University of Edinburgh, he was the first in Great Britain to lecture on the whole subject of medical psychology as distinguished from technical lectures on insanity. He promulgated the law of the reflex action of the brain, and in my opinion anticipated some of the generalisations of Spencer and Darwin. He was a daring speculator and thinker. He was not afraid of startling conclusions, tried to include all mental phenomena whatever, in animals and man, in health and disease, within his generalisations, and beyond any doubt was the most suggestive writer on the subject of his time. Next came Maudsley, who by his "Physiology and Pathology of Mind," his lectures on the "Relations Between Mind and Body," his "Body and Will," and his "Natural Causes and Supernatural Seemings," created a new literary era in our department. His eloquence, his extraordinary force, his boldness of thought and speech, his philosophic insight, place him *facile princeps* among the living masters of the department. His writings are as fascinating as the best stories. Ireland has lately given us a series of admirable studies of the effects of morbid mentalisation, chiefly from the historical and hereditary point of view, in the "Blot upon the Brain" and the "Ivory Gate." Mercier, in his "Nervous System and the Mind," has also advanced and definitized our ideas on the subject. This school has in Germany and America, passed first into the domain of the "Physiological Psychology" of Wundt and Ladd, and more lately into the experimental psychology of Galton, Fechner, Herbart, Ziehen, and Münsterberg; those together form the second group that have advanced our subject. Starting from the objective and physiological standpoint, and using the modern methods and instruments of precision of the physiologist and the physicist, their recent work reads like ordinary treatises on pure science, with mind as the "form of energy" to be investigated. To measure the exact intensity of sensations, and the rate at which sensory impulses travel along the nerves and through the gray substance of the brain; to test accurately the time that is taken for sensations to become translated into motion; the time taken for a mental effect to follow an impression on the sensory organs and for a voluntary motor act to result from such a mental impression; and to trace accurately the relations of sensations, perceptions, apperceptions and concepts, form some of the achievements of the modern "psychological laboratories." In Germany, in America, and in England this mode of accurate study of the relationships of mind and body is being actively pursued at present. This form of investigation is as yet in its infancy, and its results are uncertain in regard to the light it will shed on mental disease. But Bevan Lewis has demonstrated that in general paralysis and other forms of insanity, the mental and sensory "reaction time," that is, the time taken by impressions on the sensory organs, to be followed by mental perceptions and motor acts, is markedly abnormal. An insane man virtually sees and hears more slowly than a sane man; he cannot act so quickly after perceiving his sensations, his succeeding mental concepts are less definite and accurate, and the intensity of his sensations is less. Such knowledge adds to our previous conception of insanity as being definitely a brain disease, and will in time, no doubt, have a practical value in the diagnosis and treatment of mental disturbances.

The next and third group of men has a close relationship to the last. It consists of the pure physiologists and histologists, who, in their study of the intimate structure and functions of the living body, had to investigate those of the brain, and finding that mind was one of those brain functions, they enquired into and expounded it, not from the introspective point of view, but in relation to all the other cerebral functions. Carpenter's chapter on the functions of the brain in his "Physiology" and his work on "Mental Physiology" are still most readable and instructive, while the physiological and histological work of Lockhart Clarke, Stricker, Hitzig, Fritz, Ferrier, Obersteiner, Meynert, Golgi, Bevan Lewis, Cajal, and a host of others, has immensely advanced our knowledge of the mechanism and working of the brain. We cannot rightly understand function till we know the structure and mechanism through which this function is manifested, any more than we can know electricity except through apparatus and matter.

